# P05-17 Assessing the impact of care-physical activity initiatives for people with a low socioeconomic status on healthcare utilisation: an exploratory study

**DOI:** 10.1093/eurpub/ckac095.084

**Published:** 2022-08-29

**Authors:** Lisanne Mulderij, Annemarie Wagemakers, Kirsten Verkooijen, Maria Koelen, Stef Groenewoud

**Affiliations:** 1 Social Sciences Department, Group Health and Society, Wageningen University & Research, Wageningen, The Netherlands; 2 Radboud Institute for Health Sciences, Scientific Centre for Quality of Healthcare, Radboud University Medical Centre, Nijmegen, The Netherlands

**Keywords:** Lifestyle intervention, Physical activity, Low socioeconomic status, Health promotion, Healthcare utilisation

## Abstract

**Background:**

Care-physical activity (care-PA) initiatives are being implemented in the Netherlands to stimulate the health of citizens with a low socioeconomic status (SES), with the aim of reducing health inequality and healthcare utilisation. A two-year care-PA initiative specifically developed for citizens with a low SES, X-Fittt 2.0, was offered free of charge to participants, and included 12 weeks of intensive guidance and sports sessions, and 21 months of aftercare. As the impact of care-PA initiatives on healthcare utilisation has not yet been studied, our research question was: ‘What is the impact of participation in a care-PA initiative on the healthcare utilisation of citizens with a low SES?’.

**Methods:**

We studied the healthcare utilisation of 44 former participants of X-Fittt 2.0, focussing on general practitioner care, pharmaceutical care, hospital care, paramedical care, medical aids and mental healthcare. We compared utilisation intensity (number of healthcare claims) during the two years before participation in X-Fittt 2.0 (period 1) with utilisation intensity during the two years after initial participation (period 2) using paired t-tests.

**Results:**

As expected, utilisation intensity increased significantly for paramedical care for non-chronic disorders after participation. No differences in utilisation intensity were observed for the other healthcare categories. Furthermore, although it was not the core focus of this study, our results show that people with a low SES tend to have higher healthcare utilisation than those with a higher SES.

**Conclusions:**

This exploratory study, which is unique for its use of healthcare claims data for participants of a care-PA initiative, shows a statistically significant increase in utilisation intensity for paramedical care (supplementary healthcare insurance for non-chronic conditions) in period 2 compared with period 1, as expected. This increase is likely caused by a greater utilisation intensity for physiotherapy, which covers respectively 95% and 92% of the paramedical healthcare claims of the supplementary healthcare insurance in periods 1 and 2. The design of our study can be used as a template for future research that aims to study health care utilisation over a longer time period. The results of this and future studies can be used to improve health policies.

